# Impact of Modified Competition Formats on Physical Performance in Under-14 Female Volleyball Players: The Role of Biological Maturity

**DOI:** 10.3390/sports13110390

**Published:** 2025-11-05

**Authors:** Ricardo André Birrento-Aguiar, Francisco Javier García-Angulo, Lucas Leonardo, José Manuel Palao-Andrés, Enrique Ortega-Toro

**Affiliations:** 1 Faculty of Sports Sciences, University of Murcia, 30720 Santiago de la Ribera, Spain; ra.birrentoaguiar@um.es (R.A.B.-A.); eortega@um.es (E.O.-T.); 2 Human Movement and Sports Science (HUMSE), Faculty of Sports Sciences, University of Murcia, 30720 Santiago de la Ribera, Spain; 3 Sports Performance Analysis Association (SPAA), Faculty of Sports Sciences, University of Murcia, 30720 Santiago de la Ribera, Spain; 4 Laboratory of Sport Pedagogy, Faculty of Physical Education and Physiotherapy, Federal University of Amzonas, Manaus 69067-005, Brazil; 5 Health, Exercise Science and Sport Management, University of Wisconsin-Parkside, Kenosha, WI 53144, USA

**Keywords:** modified rules, kinematic variables, maturity performance

## Abstract

The present study aimed to examine the influence of different competition models on the physical performance of under-14 female volleyball players, attending to biological maturity development. A quasi-experimental design was conducted involving 29 regional-level players (mean percentage of predicted adult height [PAH] = 95.38%). Three tournament formats were implemented: Standard Tournament (ST) 1 followed official regulations; Modified Tournament 1 (MD1) featured modified rules including a reduced net height (from 2.10 m to 2.00 m), prohibition of jump serves, and a maximum of two consecutive serves per rotation; and Modified Tournament 2 (MD2) included all prior modifications alongside a reduced court size (from 9 × 9 m to 8 × 8 m). Performance metrics analyzed included the number of accelerations, decelerations, impacts, and jumps (total count, G-force, take-off, and landing characteristics). Measures were gathered using a local positioning system (LPS) device based on UWB technology and an inertial measurement (IMU; WIMU PROTM, Real Track Systems, Almeria, Spain). Significant differences were observed between the tournaments, with Modified Tournament 1 (MD1) and Modified Tournament 2 (MD2) showing higher values in accelerations (*p* = 0.005), decelerations (*p* = 0.005), impacts (*p* < 0.01), and jumps (*p* < 0.01) compared to Standard Tournament. Notably, the greatest improvements were found between Standard Tournaments (ST) and Modified Tournament 2 (MD2). These findings suggest that modified competition formats enhance kinematic performance in under-14 female volleyball players. The results support the need for age- and maturity-appropriate adjustments to competition regulations in youth volleyball.

## 1. Introduction

In team sports, such as volleyball, the interaction between teammates, the environment, and tasks is an essential characteristic [1,2,3]. For many years, youth sport has been governed by rules and regulations derived from professional sport, without fully considering the differences between children, adolescents, and adults [4,5,6]. However, various studies have demonstrated that young players prefer practicing with adapted equipment and reduced dimensions, which increases their participation, enhances their perception of self-efficacy, and promotes the acquisition of technical–tactical skills [7,8].

In volleyball, federations and educational bodies have implemented adaptations such as mini volleyball [7,9] scaling structural aspects such as court size, net height, and ball size. Despite these adaptations, from U-12 categories onwards, the game progressively resembles the adult version, mainly through increasing net height and court size, although there is insufficient evidence to validate the adequacy of these modifications for each age group and maturity level [10,11].

The manipulating of the net height and court size has direct effects on ball trajectory, interception time, and the quality of technical–tactical actions such as reception, attack, serving, and spiking [12,13]. A higher net height may benefit the time available for defensive actions but makes serving and spiking more challenging. Similarly, larger courts increase interception difficulty, whereas reduced courts promote game continuity and technical learning. Consolidating these effects in a single section allows for a clearer understanding of how structural adaptations influence both physical and technical–tactical performance.

Compared with sports such as football and basketball, where rule modifications and their impact on participation, physical performance, and psychological perception have been widely studied [6,14,15], volleyball still presents questions regarding the real influence of adaptations such as reducing net height or court size. Experimental studies confirming these effects in real competitive contexts are limited [16].

Biological maturity also plays a crucial role in youth volleyball. Current competitive categories are established solely based on chronological age, without considering biological maturation status [17,18,19,20]. This creates physical imbalances among players with different growth rates, a situation already identified in sports such as football, where use bio-banding to group players according to their maturation status [21,22]. Implementing maturation-based grouping allows for a more balanced game, encourages participation, and reduces the risk of early dropout due to frustration or lack of interest in late-maturing players [23,24,25].

Recent analytical approaches developed in youth football have highlighted the importance of integrating task constraints with contextualized models to better understand players’ performance. These frameworks emphasize that contextual factors, including rally length, player rotation and role, and set duration, should be analyzed alongside external-load metrics to capture the true dynamics of competition. Applying such a perspective to volleyball could enhance the understanding of how environmental and structural adaptations interact with players’ physical and technical–tactical responses under realistic match conditions [17,18].

In volleyball, a study involving U-14 female players demonstrated that reducing net height, decreasing court size, and limiting the serve—prohibiting jumping serves and allowing a maximum of two serves per rotation—increased side-out effectiveness, the number of attacks and blocks, game continuity, as well as physical involvement and players’ perceptions of self-efficacy and satisfaction [25]. Additionally, a longitudinal study involving U-13 and U-14 female players analyzed the evolution of jump performance and postural control as a function of age and maturation, highlighting the importance of adjusting training and competition formats according to maturational development [26].

It has also been found that in competitive youth volleyball, adjusting net height in reduced games (4 vs. 4) showed that a lower net favours serving, whereas an official-height net improves defence, evidencing that manipulating net height and court size influences technical–tactical performance [27]. Another study compared methods for predicting maturation in a U-17 female team, finding that 69% of players presented early maturation, indicating that adjustments based on maturity are crucial to avoid biases in selection and training load [28].

For all these reasons and considering both biological maturity and the effects of competition models, the aim of the present study was to examine the influence of different competition models on the physical performance of under-14 female volleyball players, attending to biological maturity development.

## 2. Materials and Methods

### 2.1. Sample

The participant group comprised 29 female volleyball players from the under-14 level, all competing in regional-level amateur club tournaments. The cohort presented the following average characteristics: age 13.4 ± 0.68 years; height 1.63 ± 0.06 m; body mass 55.5 ± 7.9 kg; an average of 2.85 ± 0.31 weekly training sessions; mean session duration of 1.37 ± 0.44 h; and a mean volleyball practice experience of 3.21 ± 0.85 years. At the point of data collection, all players had already undergone pubertal development. Prior to participation, the legal guardians of all athletes were duly informed of the study’s procedures and provided written informed consent in accordance with ethical requirements.

Throughout the study, the players took part in three tournaments, each employing a different competition format: official rules, modified rules option 1, and modified rules option 2. The study received approval from the ethics committee of the university affiliated with the research team (reference ID 2556/2019 approval date: 11 March 2020).

### 2.2. Design

A quasi-experimental design was employed to analyze the impact of altering specific game parameters: net height, type of serve, restrictions on consecutive serves per player, and court dimensions. The independent variable corresponded to the competition format, defined by three conditions: Standard Tournament (ST) (serving as the control tournament) and two variations incorporating modified rules, Modified Tournament 1 (MD1) and Modified Tournament 2 (MD2).

The tournaments were conducted sequentially (ST → MD1 → MD2), with each team playing only two matches per tournament. This design introduces potential order effects, opponent variability, differences in set/rally duration, and temporal confounding, which were not modelled or controlled.

The primary distinctions between the official and modified formats were the following: net height (2.10 m in ST vs. 2.00 m in MD1 and MD2), court size (9 × 9 m un ST vs. 8 × 8 m in MD2, referring to side-by-side dimensions), and serving constraints (unrestricted in ST vs. a maximum of two consecutive serves per rotation and prohibition of jump serves in the MD1 and MD2). Upon reaching the serve limit, teams were required to rotate positions.

MD1 implemented a reduction in net height from 2.10 m to 2.00 m, prohibition of jump serves, and a maximum of two consecutive serves per rotation. MD2 included the same net height reduction, the same serving restrictions, and a reduction in court size from 9 × 9 m to 8 × 8 m. Throughout all tournaments, athletes participated with their habitual teams.

### 2.3. Procedure and Materials

The study consisted of three different competitions. Each team played two matches per tournament. In each tournament, the following kinematic data were collected: (a) accelerations and decelerations (number of accelerations, number of decelerations, maximum acceleration (m/s^2^), maximum deceleration (m/s^2^), number of high accelerations; number of high decelerations, distance covered in high acceleration, distance covered in high deceleration); (b) impacts (total of impacts; number of 0–3 g impacts; number of 3–5 g impacts; number of 5–8 g impacts and >8 g impacts); and (c) jumps (number of jumps, average take-off (g), average landing (g), number of high take-offs, number of high landings, number of 3–5 g landing; number of 5–8 g landing; >8 g landing; 0–3 g take-off; 3–5 g take-off and 5–8 take-off).

Measures were gathered using a real-time motion tracking system that included a local positioning system (LPS) based on UWB technology and an inertial measurement unit (IMU; WIMU PRO™, RealTrack Systems, Almería, Spain) in an indoor basketball court. Crucial implementation details such as anchor placement, calibration, sampling rate, and potential occlusions were standardized according to manufacturer recommendations. Data extraction was performed using SPRO Version 1.0.0 Compilation:989 software (RealTrack Systems, Almería, Spain). Although previously validated in other contexts [28], specific validation for indoor volleyball was not performed.

**Figure 1:**
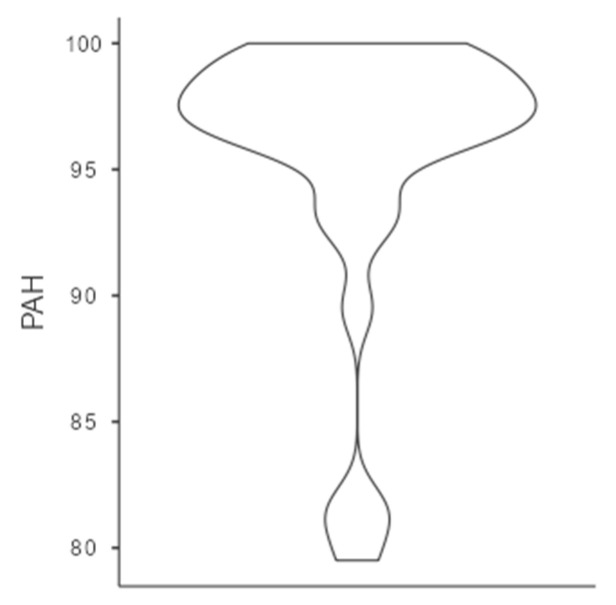
Distribution of the %PAH variable.

Before the tournament, somatic maturation data were collected. The weight and height of players were measured using a standard stadiometer (actual model verified and corrected from Tanita BF-522W) and a calibrated scale. Parental heights were self-reported. The weight and height of the players were measured together with the height of both parents. The Tanita stadiometer (Tanita BF-522W, Tokyo, Japan) was used for this measurement. With this information, the maturational age was calculated through the percentage of predicted adult height (%PAH), using the Khamis–Roche formula [29,30]. The %PAH variable was used as a continuous measure to analyze how maturational age moderated the intervention. The distribution of the %PAH data can be seen in Figure 1.

### 2.4. Data Analysis

This study adopted a Bayesian approach as its main evidentiary framework. Inferences were based on model comparison using Bayes factors (BF_10_) and Bayesian odds ratios, which quantify the relative evidence in favour of the alternative hypothesis (H_1_) vs. the null hypothesis (H_0_). Frequentist analyses are included only as supplementary information on the magnitude and precision of the observed effects.

The normality of the data distribution was assessed. For variables with a normal distribution, a repeated-measures ANOVA was performed; for variables with a non-normal distribution, the Friedman test was applied. Effect size was calculated using partial η^2^. In addition, a Bayesian repeated-measures ANOVA was conducted. For the interpretation of differences, the Bayes factor (BF_10_) was used. Bayesian post hoc comparisons between tournaments were conducted to evaluate specific differences, using a Bayesian approach with multiple comparison correction [31]. In the event of discrepancies between Bayesian and frequentist metrics, interpretative priority was given to the Bayesian result. Posterior odds (PO) were used as the main indicator. To interpret this analysis, the following classification scale was employed: values below 1/100 indicate extreme evidence supporting H0; between 1/100 and less than 1/30 represent very strong evidence for H0; from 1/30 to less than 1/10 denote strong evidence for H0; from 1/10 to less than 1/3 indicate moderate evidence for H0; from 1/3 to less than 1 correspond to ambiguous evidence in favour of H0; values between 1 and 3 suggest anecdotal evidence supporting H1; those greater than 3 up to 10 indicate moderate evidence for H1; greater than 10 up to 30 reflect strong evidence for H1; greater than 30 up to 100 show very strong evidence for H1; and values exceeding 100 demonstrate extreme evidence in support of H1 [32].

A within-subject repeated-measures moderation analysis was performed to determine whether players’ maturational age moderated the impact of the rule modification (i.e., differences observed between tournaments). The association between the predictor and outcome variables was evaluated by examining their interaction with the continuous moderator variable (W1) using a simple moderation framework [33]. Accordingly, an ordinary least squares regression analysis was conducted employing the SPSS macro MEMORE v2.1 [33]. Considering the continuous nature of the variables, the Johnson–Neyman procedure was applied to identify regions of significance—that is, specific ranges of maturational age where the intervention yielded statistically significant effects [34].

## 3. Results

The results in Table 1 reflect the differences found in the variables linked to accelerations and decelerations between tournaments.

Significant differences were found for the variables number of accelerations, number of decelerations, and maximum deceleration speed. For the number of accelerations, the posterior odds indicate anecdotal evidence between ST and MD2 (PO = 2.693), as well as between MD1 and MD2 (PO = 2.556), with a higher number of accelerations observed in MD2. In the number of decelerations, anecdotal differences were found between ST and MD2 (PO = 2.807) and between MD1 and MD2 (PO = 2.430). For maximum deceleration speed, anecdotal evidence was found between ST and MD1 (PO = 2.646), in favour of MD1. Although the posterior odds indicated only anecdotal evidence in each case (PO: range: 2.43–2.80), the results followed a consistent trend, with higher values observed in the modified formats—particularly MD2.

**Figure 2:**
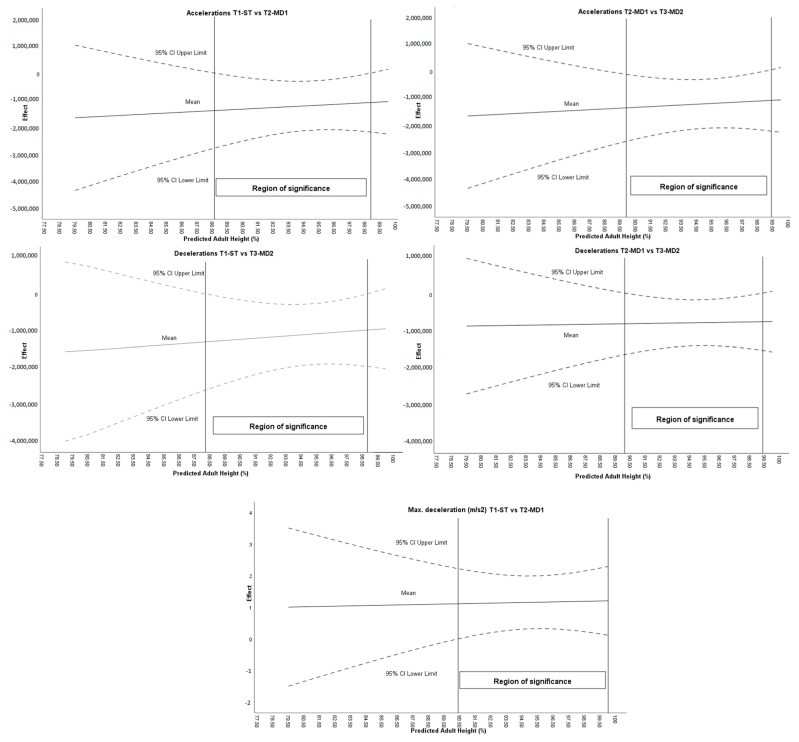
Moderating effect of maturational age on acceleration-related variables.

Figure 2 shows how maturational age conditions the effect of the intervention on the acceleration variables.

Regarding the moderation analysis, the Johnson–Neyman procedure revealed specific ranges of maturational stages that moderated the effect on deceleration and maximum deceleration (Figure 2). The moderating effect for accelerations between tournaments 1–2 was found between 88.62 and 98.88% PAH and between MD1 and MD2 for a %PAH of 89.88–99.39. For the deceleration’s variable, maturational age conditioned the intervention between ST and MD2, specifically in the range 88.20–98.83% PAH, and between MD1 and MD2 in the range 90.10–99.37% PAH. For maximum deceleration, %PAH conditioned intervention between ST and MD1 for players with a %PAH higher than 90.37%. For all other variables listed in Table 1, the Johnson–Neyman analysis revealed no regions of significance, indicating that age moderated the observed effects.

Table 2 shows the differences between the different tournaments in the impact variables.

Significant differences were found in the variables total number of impacts, and number of impacts between 0 and 3 g, 3 and 5 g, and higher than 8 g. For the total number of impacts, strong evidence was found between ST and MD2 (PO = 15.684), and anecdotal evidence between Tournament MD1 and MD2 (PO = 1.167), with MD2 showing the highest number of impacts. In the number of impacts between 0 and 3 g, strong evidence was found between ST and MD2 (PO = 16.074), and anecdotal evidence was found between MD1 and MD2 (PO = 1.128), again with MD2 having the highest number of impacts. Regarding the number of impacts between 3 and 5 g, moderate evidence was found between ST and MD2 (PO = 4.470), and anecdotal evidence was found between MD1 and MD2 (PO = 2.719). Anecdotal evidence was also found in favour of MD2 for the number of impacts higher than 8 g (PO = 1.145). Although the posterior odds indicated only anecdotal evidence in most pairwise comparisons involving MD1 and MD2 (PO range: 1.128–2.719), the results revealed a consistent trend, with MD2 producing the highest number of impacts across all intensity ranges.

**Figure 3:**
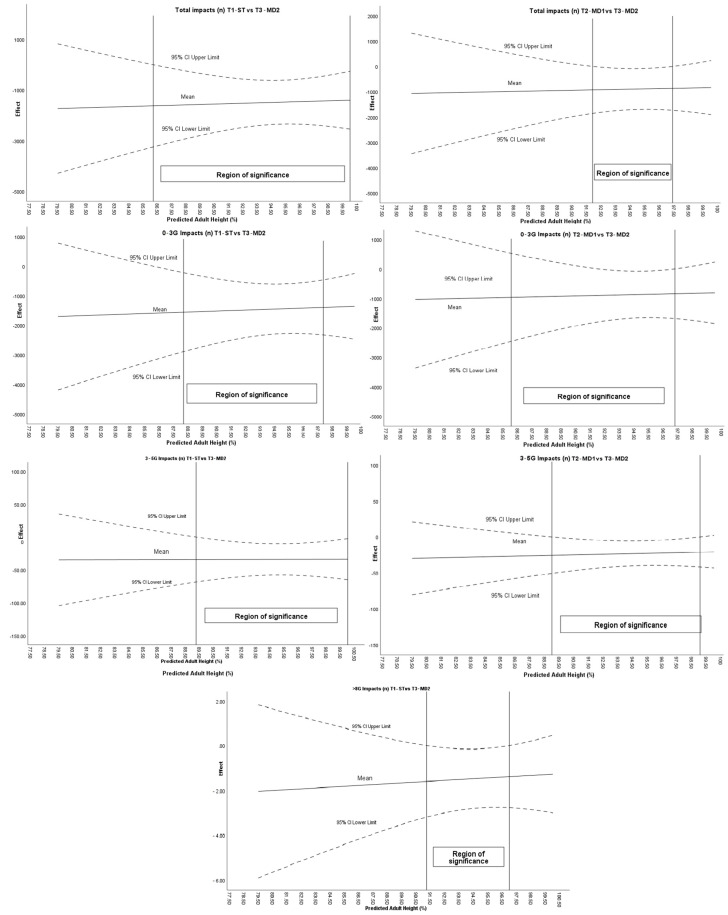
Moderating effect of maturational age on impact-related variables.

Figure 3 shows how the age at maturity conditions the effect of the intervention on the variables associated with the impacts of the intervention.

Regarding the moderation analysis, the Johnson–Neyman procedure identified specific ranges of maturational stages that moderated the total number of impacts, 0–3 G impacts, 3–5 G impacts and >8 impacts (Figure 3). The moderating effect for total impacts between tournaments 1–3 was found in players with %PAH greater than 86.19% and between MD1 and MD2 for a %PAH of 91.90–97.38. For the 0–3 g impacts variable, maturational age conditioned the intervention between ST and MD2, specifically with a %PAH higher than 86.06%, and between MD1 and MD2 in the range 92.03–97.27%PAH. For 3–5 G impacts, %PAH conditioned intervention between ST and MD2 for players with a %PAH higher than 89.24% and between MD1 and MD2 for a %PAH 88.99–99.07%. In the >8 G impacts, the %PAH moderates the intervention between ST and MD2, specifically with players with a %PAH between 91.22 and 96.97%.

Table 3 shows the differences found between the designed tournaments in the variables linked to jumping.

Significant differences were found in the number of jumps, average take-off, average landing, number of landings between 3 and 5 g, and number of take-offs between 0 and 3 g. The posterior odds indicate strong evidence of differences in the number of jumps between ST and MD2 (PO = 16.684), and moderate evidence between MD1 and MD2 (PO = 4.866). Regarding average take-off, moderate evidence was found when comparing ST and MD1 (PO = 6.463) and ST and MD2 (PO = 8.457), with ST showing the lowest values. For average landing force, moderate evidence was observed between ST and MD1 (PO = 4.307), and anecdotal evidence was found between ST and MD2 (PO = 1.772). In the variable of landings between 3 and 5 G, strong evidence was found when comparing ST with MD1 (PO = 16.802), and moderate evidence was found when comparing ST with MD2 (PO = 7.883). Finally, for take-offs between 0 and 3 G, moderate evidence was observed when comparing ST with MD2 (PO = 7.079), and anecdotal evidence was found between MD1 and MD2 (PO = 2.783). The results indicate that the modified formats, especially MD2, promoted greater motor engagement in under-14 players, with more jumps and moderate-intensity landings (3–5 g). This suggests and increase in the frequency of explosive actions with lower overload risk, creating a safer and more simulating environment for physical development.

**Figure 4:**
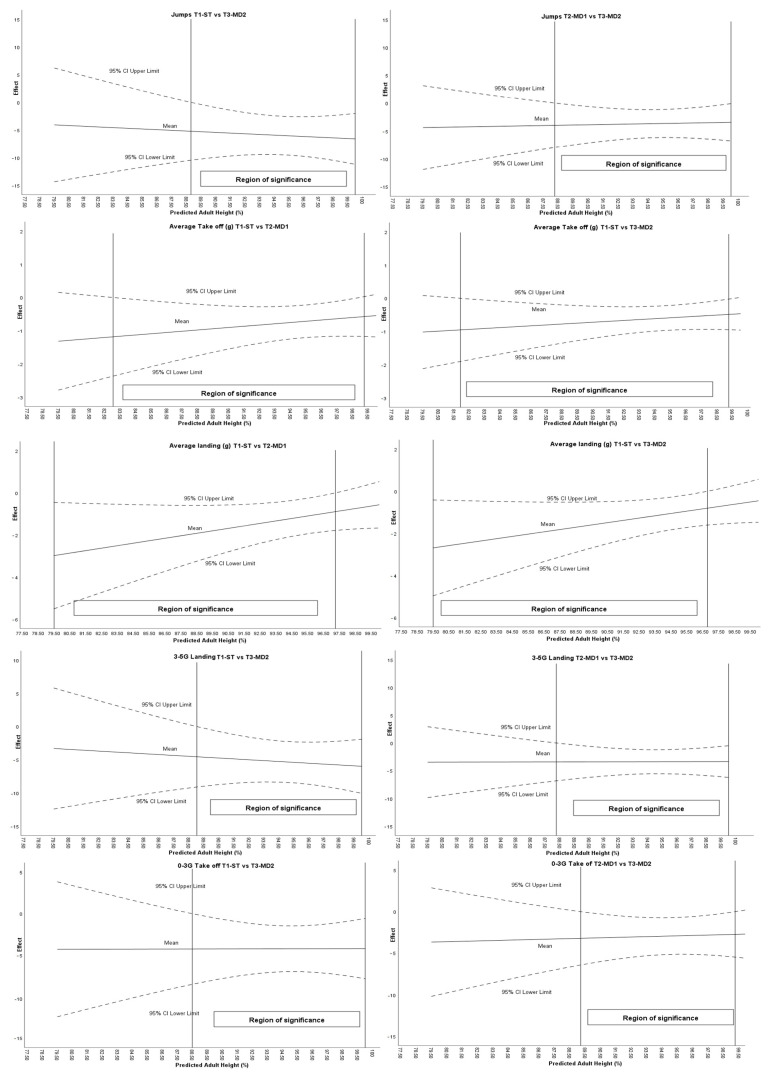
Moderating effect of maturational age on the intervention on jumping-related variables.

Figure 4 reflects the moderating effect of maturational age on the intervention on jumping-related variables.

It was found that maturational age influenced the effect of the intervention on the jumps (ST vs. MD1: >88.80% PAH; MD1 vs. MD2: >88.26% PAH); average take-off (ST vs. MD1: 83.03–98.95% PAH); average landing (ST vs. MD1: <97.25% PAH; ST vs. MD2: <96.80% PAH); 3–5 G landing (ST vs. MD1: >89.03% PAH; MD1 vs. MD2: >88.26% PAH); and 0–3 g take-off (ST vs. MD2: >88.48% PAH; MD1 vs. MD2: 89.20–99.25% PAH).

## 4. Discussion

This study investigated the impact of different competition models, including modifications in net height, court size, and serving restrictions, on the physical performance of under-14 female volleyball players and explored how biological maturity (%PAH) moderated these effects. Overall, the results showed that the modified formats, particularly MD2, elicited greater physical engagement, with higher numbers of accelerations, decelerations, jumps, and impacts at various intensities. Additionally, it examined how biological maturity, measured as percentage of predicted adult height (%PAH), moderated these effects. The results align with prior research in youth sports, reinforcing the significance of tailoring competition formats to players’ developmental stages [35,36].

The findings revealed that the modified rules implemented in MD2 (lower net height, smaller court dimensions, and serve restrictions) elicited greater physical engagement, as evidenced by an increased number of accelerations, decelerations, jumps, and impacts at various intensities. These outcomes align with previous research indicating that reducing structural constraints accelerates the pace of play and enhances motor involvement in youth sport contexts [25].

Specifically, MD2 promoted more frequent but less intense actions (e.g., 0–3 g impacts or low-force take-offs), which may facilitate motor learning while reducing fatigue and injury risk. This provides further support for the pedagogical value of modified game formats in enhancing technical–tactical engagement among young players. This trend is consistent with the literature on small-sided games (SSG) in volleyball, where formats involving fewer players and reduced playing areas have been shown to increase physical load, game tempo, and technical–tactical involvement without compromising the overall volume of activity [10,11].

Biological maturation significantly moderated several variables, particularly those related to higher-intensity actions. For instance, in parameters such as peak deceleration speed or impacts exceeding 8 g, the effects were more pronounced in athletes with a percentage of predicted adult height (%PAH) above ~90%. This suggests that more biologically mature players respond differently to rule modifications compared to their less mature peers, underscoring the relevance of integrating maturation metrics in competition design. This perspective is supported by previous work examining how maturity status influences physical responses to changes in rules or formats within youth sports [36].

From a pedagogical standpoint, the observed increase in the number of jumps and landings—particularly in MD2—indicates enhanced engagement in key volleyball actions. Within physical education and early sports development contexts, recent studies on SSG-based interventions in young players have demonstrated that manipulating space and rules can improve explosive power (e.g., blocking or attacking jumps) as well as technical and tactical behaviours [37].

Taken together, these findings highlight the value of modified competition models for promoting physical and technical–tactical engagement in youth volleyball, particularly when considering the players’ maturational stage. The results suggest that carefully designed modifications can optimize training load, motor involvement, and skill development, while reducing the risk of overload.

Taken together, these results—alongside prior evidence concerning self-perception and motivation in youth volleyball adaptations—support the continued implementation of modified game formats. Educational frameworks such as the dual convergent model [38], which integrates both technical–tactical and psychosocial dimensions, find strong justification within this type of structural adaptation. Such approaches appear especially valuable in optimizing learning processes during early developmental stages.

In terms of limitations, it should be noted that participants did not have prior experience practicing under the modified rules before the tournaments. This may have influenced their responses, particularly in more complex actions requiring adaptation to the new competition format. Future studies should consider a familiarization period to better capture the potential benefits of rule modifications.

## 5. Conclusions

In conclusion, this study underscores the importance of implementing adapted competition formats to better align physical demands with the biological maturation of youth volleyball players. Our results indicate that players within specific maturity ranges—generally between ~88–99% of predicted adult height (%PAH), depending on the variable—benefited most from the modified formats, particularly MD2, with increases in accelerations, decelerations, jumps, and impacts. Incorporating maturity-related metrics into tournament structuring and athlete progression pathways has the potential to promote more equitable, developmentally sensitive environments that support both performance enhancement and sustained engagement in the sport. Such adaptations may help mitigate physical disparities and encourage age-appropriate motor engagement, while hypotheses regarding safer participation and less intense actions require further investigation using direct measures of injury, soreness, or wellness. Future research should investigate the longitudinal effects of these modifications, particularly their influence on psychological well-being, motivation, and the development of technical–tactical competencies across different stages of maturation.

## Figures and Tables

**Table 1:** Differences between tournaments for variables related to accelerations and decelerations.

B	ST	MD1	MD2	*p*-Value	Effect Size	BF_10_	Post Hoc
	M ± SD [IC 95%]	M ± SD [IC 95%]	M ± SD [IC 95%]				
Accelerations (*n*)	495 ± 191 [422; 568]	530 ± 179 [461; 600]	616 ± 217 [530; 702]	0.005	0.182	7.78	T1 < T3 T2 < T3
Decelerations (*n*)	456 ± 181 [387; 525]	487 ± 171 [421; 553]	566 ± 203 [486; 647]	0.005	0.182	7.62	T1 < T3 T2 < T3
Max. Acceleration (m/s^2^)	5.06 ± 1.75 [4.35; 5.68]	5.32 ± 1.44 [4.77; 5.89]	4.85 ± 1.4 [4.30; 5.41]	0.641	0.024	0.192	
Max. deceleration (m/s^2^)	4.09 ± 1.19 [−4.57; −3.66]	5.22 ± 1.44 [−5.80; −4.68]	4.75 ± 1.34 [−5.28; −4.22]	0.008	0.143	6.51	T1 > T2
High acceleration (*n*)	17 ± 13.8 [11.8; 13.8]	16.9 ± 12.3 [11.8; 21.4]	16.9 ± 12.6 [11.9; 21.9]	0.717	0.000	0.108	
High deceleration (*n*)	16.4 ± 10.4 [12.4; 20.3]	16.9 ± 11.4 [12.3; 21.2]	17.1 ± 11.8 [12.4; 21.7]	0.886	0.000	0.016	
Distance covered in high acceleration (m)	24.8 ± 16 [18.1; 30.3]	25.4 ± 21.2 [16.7; 33.1]	22.8 ± 17.1 [16; 29.6]	0.895	0.009	0.129	
Distance covered in high deceleration (m)	27.1 ± 14.3 [20.6; 31.4]	25.9 ± 19.2 [17.9; 32.8]	24.7 ± 20.2 [16.7; 32.7]	0.618	0.007	0.125	

ST = Standard Tournament; MD1 = Modified Tournament 1; MD2 = Modified Tournament 2.

**Table 2:** Comparison between tournaments on impacts.

	ST	MD1	MD2	*p*-Value	Effect Size	BF_10_	Post Hoc
	M ± SD [IC 95%]	M ± SD [IC 95%]	M ± SD [IC 95%]				
Total impacts (n)	4029 ± 2541 [3063; 4996]	4521 ± 2493 [3554; 5487]	5551 ± 3425 [4197; 6906]	<0.001	0.238	34.82	T1 < T3 T2 < T3
0–3 g impacts (n)	3929 ± 2474 [2988; 4870]	4409 ± 2426 [3468; 5350]	5409 ± 3326 [4093; 6725]	<0.001	0.237	33.81	T1 < T3 T2 < T3
3–5 g impacts (n)	78.7 ± 62 [55.1; 102]	89.6 ± 58.9 [66.7; 112]	115 ± 87.5 [80.7; 150]	0.022	0.219	20.15	T1 < T3 T2 < T3
5–8 g impacts (n)	20.3 ± 36.3 [6.5; 34.1]	19.8 ± 16.8 [13.3; 26.3]	23.7 ± 20.1 [15.7; 31.6]	0.010	0.006	0.124	
>8 g impacts (n)	1.52 ± 2.6 [0.53; 2.51]	2.43 ± 3.26 [1.16; 3.69]	3.07 ± 3.87 [1.54; 4.61]	0.022	0.121	1.63	T1 < T3

ST = Standard Tournament; MD1 = Modified Tournament 1; MD2 = Modified Tournament 2.

**Table 3:** Comparison between tournaments in the variables associated with jumps.

	ST	MD1	MD2	*p*-Value	Effect Size	BF_10_	Post Hoc
	M ± SD [IC 95%]	M ± SD [IC 95%]	M ± SD [IC 95%]				
Jumps (n)	7.45 ± 10.6 [3.43; 11.5]	9.86 ± 10.3 [5.87; 13.8]	13.9 ± 13.8 [8.41; 19.4]	0.002	0.254	57.34	T1 < T3 T2 < T3
Average take-off (g)	1.41 ± 0.94 [1.05; 1.77]	2.14 ± 0.97 [1.78; 2.5]	2.05 ± 0.68 [1.78; 2.32]	<0.001	0.236	56.58	T1 < T2 T1 < T3
Average landing (g)	2.71 ± 1.72 [2.05; 3.36]	3.81 ± 1.31 [3.3; 4.32]	3.69 ± 1.07 [3.27; 4.11]	0.121	0.198	28.67	T1 < T2 T1 < T3
High take-off (n)	1.93 ± 3.93 [0.43; 3.42]	3 ± 4.68 [1.18; 4.82]	3.85 ± 4.66 [2.01; 5.70]	0.002	0.140	2.5	
High landing (n)	1.59 ± 2.51 [0.63; 2.54]	1.96 ± 2.85 [0.86; 3.07]	2.33 ± 3.01 [1.14; 3.53]	0.267	0.031	0.20	
3–5 g landing	5.86 ± 8.79 [2.52; 9.20]	7.89 ± 8.05 [4.77; 11]	11.6 ± 11.4 [7.05; 16.1]	0.002	0.270	92.67	T1 < T3 T2 < T3
5–8 g landing	1.45 ± 2.29 [0.57; 2.32]	1.57 ± 2.08 [0.76; 2.38]	2.11 ± 2.79 [1.01; 3.22]	0.421	0.033	0.214	
<8 g landing	0.138 ± 0.44 [−0.03; 0.31]	0.40 ± 0.93 [0.04; 0.77]	0.22 ± 0.42 [0.05; 0.4]	0.272	0.075	0.520	
0–3 g take-off	5.52 ± 7.13 [2.80; 8.23]	6.83 ± 6.88 [4.19; 9.52]	10 ± 10.6 [5.83; 14.2]	0.002	0.213	17.16	T1 < T3 T2 < T3
3–5 g take-off	1.90 ± 3.93 [0.40; 3.39]	2.82 ± 4.34 [1.14; 4.5]	3.67 ± 4.43 [1.91; 5.42]	0.002	0.121	1.55	
5–8 take-off	0.03 ± 0.18 [−0.04; 0.1]	0.18 ± 0.48 [0; 0.3]	0.18 ± 0.58 [−0.03; 0.4]	0.422	0.040	0.274	

ST = Standard Tournament; MD1 = Modified Tournament 1; MD2 = Modified Tournament 2.

## Data Availability

The data presented in this study are available on request from the corresponding author due to ethical restrictions. The dataset includes information collected from underage participants, and to protect their privacy and confidentiality, direct sharing is not permitted.
